# Characteristics of an underground stope channel supplied by atmospheric precipitation and its water disaster prevention in the karst mining areas of Guizhou

**DOI:** 10.1038/s41598-023-43209-4

**Published:** 2023-09-23

**Authors:** Xianzhi Shi, Weiqiang Zhang

**Affiliations:** 1 School of Resources and Earth Science, China University of Mining and Technology, Xuzhou, China; 2 Guizhou Yuxiang Mining Group Investment Co. Ltd., Bijie, China

**Keywords:** Natural hazards, Hydrogeology

## Abstract

Atmospheric precipitation floods mining areas, which seriously affects the safe production of coal mines. However, research on the mechanism underlying precipitation supplying coal mines, particularly in karst landform areas, remains far from sufficient. Based on the collection of a large amount of geological and hydrogeological mining data and some data related to mine atmospheric precipitation and mine water inflow, the channels of atmospheric precipitation supplying mines in the main coal-producing areas of Guizhou, China, are systematically analysed and studied. They are divided into weathering zone fractures, mining fractures, water diversion faults, water diversion collapse columns and karst channels. Recharge channels have the characteristics of surface infiltration, pipeline flow and layered recharge, as well as self-healing after being filled by surface loess and other materials. The supply of atmospheric precipitation to the coal mine stope is seasonal. The mine water inflow in the rainy season is 1.2 ~ 12 times that in the dry season, with an average of 1.9 times. The supply has hysteresis. The lag time of surface infiltration, pipeline flow and layered flow is 2 ~ 4 days, within 24 h and more than 2 days, respectively. The recharge is affected by the burial depth of the coal seam and the characteristics of the combined upper roof slate. Among the mines affected by atmospheric precipitation and water disasters, some mines have carried out research on the comprehensive treatment of water disasters, implemented supplementary exploration projects such as surface hydrogeological drilling and geophysical exploration, or carried out hydrochemical research. Some mines have adopted water prevention and control projects, such as blocking ground water diversion cracks, constructing water diversion projects, adjusting the mining time of the working face, transforming the drainage system and improving the drainage capacity, to ensure the safe production of mines. This research achievement may provide a theoretical basis and practical experience for the prevention and control of atmospheric precipitation infiltration in coal mines in karst areas.

## Introduction

Surficial karst is present in coal-producing areas of southwestern China, with large undulating terrain and complex geomorphic types. The coal seams in the early mining faces of the mine are relatively shallowly buried. The average annual rainfall in this region ranges from 1073 to 1439 mm, with significant concentrated precipitation. Atmospheric precipitation is mainly concentrated from April to September, accounting for more than 80% of the annual precipitation. Affected by atmospheric precipitation, the water flowing into coal mines in this region varies significantly with seasonal atmospheric precipitation. Atmospheric precipitation is one of the main water sources for coal mine production in this region^1–7^, and at certain points, it affects the safety of coal mine production^1,2,4,7,8^.

With the integration or closure of a large number of coal mines in southwestern coal-producing areas, such as Hunan Province and Chongqing city, Guizhou Province, as the only one-billion-ton coal base in southwestern China, has become the main coal production and supply area in southwestern China and is also an important energy supply province for the west to east power transmission. The main coal-producing areas in the province are located in karst landform-developed areas, where faults are developed and water gushing from coal mines is affected to varying degrees by atmospheric precipitation^5–10^. Atmospheric precipitation causes some mining faces, areas or mines to be flooded every year. From 2005 to 2026, there were 103 water accidents, with 325 deaths^11^. From 2021 to 2022, during the rainy season in Jinsha alone, 6 pairs of mines in Guizhou Province experienced a sudden increase in underground water inflow caused by atmospheric precipitation, which affected the safety of mine production. Atmospheric precipitation has had a significant impact on the safe production and coal production volume of coal mines in karst areas of Guizhou, limiting the effective improvement in coal production capacity and the power supply in the eastern region.

Numerous scholars and engineering technicians^2,4–6,12,13^, when predicting mine water inflow and controlling and preventing mine water hazards, have found that the main source of water flowing into mines in karst mining areas is atmospheric precipitation. The research results of Zhang^8^, Shi et al.^9^, and Xu^10^ et al. suggest that atmospheric precipitation can supply mine stopes through karst channels, the limestone strata on the roof of coal-bearing strata can become supply channels for atmospheric precipitation, and faults can become supply channels for atmospheric precipitation, thereby supplying mine stopes. In the research results of Mahmud al-Islam and Hasibul Hasan^14^, the calculation of surface runoff from atmospheric precipitation and the delineation of the infiltration range play an important guiding role in studying replenishment of mine stopes by atmospheric precipitation.

The abovementioned scholars and engineering technicians^2,4–10,12,13^ have conducted extensive research on the supply of atmospheric precipitation to mine stopes, providing a large amount of technical data for studying the supply of atmospheric precipitation to mining sites. However, there is no research on the scope and amount of atmospheric precipitation infiltration under mining failure conditions, and there is a lack of systematic research on the channels supplying atmospheric precipitation. There is no classification of channels supplying atmospheric precipitation, and no scholars have studied the characteristics of various water channel replenishment. There is a lack of empirical research on controlling and preventing water hazards based on the characteristics of water-conducting channels that have been discovered via various research findings.

On the basis of analysing and studying a large amount of geological and hydrogeological mine data, as well as some typical data related to atmospheric precipitation and mine water inflow, this article classifies the supply channels of atmospheric precipitation to mines in the main coal mining area of Guizhou and investigates the characteristics of water channels and atmospheric precipitation supply to mine water. Measures for controlling and preventing atmospheric precipitation and water damage in mines are also discussed. This research achievement provides a theoretical basis and practical experience for controlling and preventing water damage from atmospheric precipitation in mines in karst landform conditions.

## Materials and methods

### Karst characteristics in the study area

Karst landforms form by the dissolution of water and account for 10% of the Earth's surface. Therefore, karst landforms are very common in nature. Karst landforms are widely distributed in China, mainly in the carbonate-exposed areas in the western provinces. The national karst landform area, including Guizhou, is approximately 0.91 × 10^6^–1.30 × 10^6^ km^2^^15^, wherein Guangxi, Guizhou and eastern Yunnan have the largest areas. Yunnan Guizhou Plateau in Southwest China is the area with the most karst landforms. There are many coal resources at the junction of Yunnan, Guizhou and Guangxi on the Yunnan Guizhou Plateau, which is one of China's coal production bases that produces hundreds of millions of tons. Furthermore, the main coal-producing areas in Guizhou are distributed in Zunyi city, Bijie city, Liupanshui city, Qianxinan Prefecture and Guiyang city; in these areas, the coal seams of the Permian Longtan Formation between the Triassic Yelang Formation (Feixianguan Formation) (T_1_) and the Permian Maokou Formation are mainly mined, and each field is located in the area of karst landform development.

**Figure 1:**
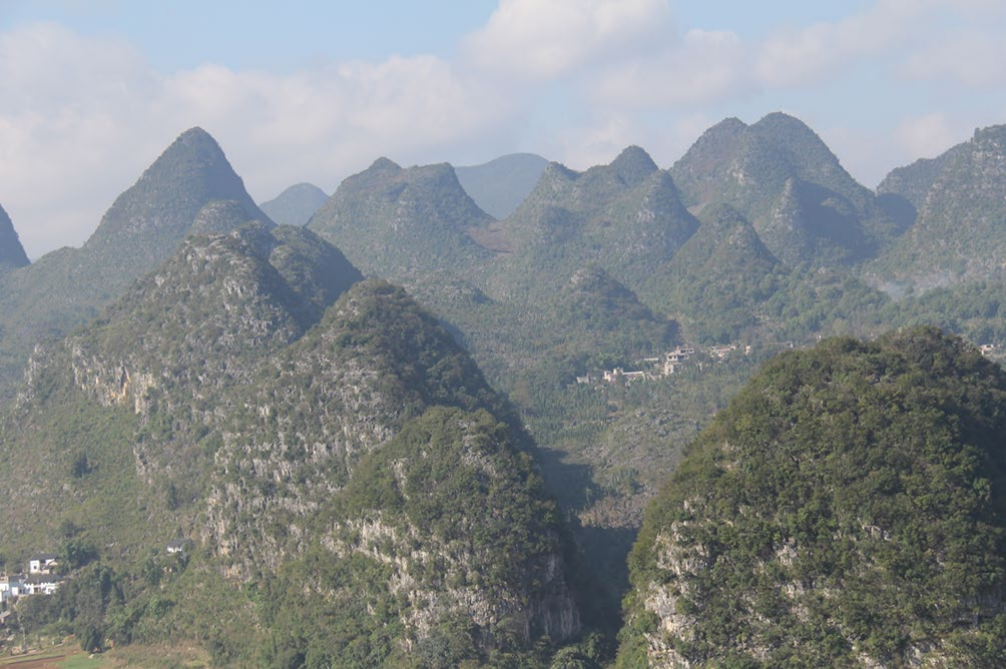
Karst Landform in Qianxinan Prefecture, Guizhou.

**Figure 2:**
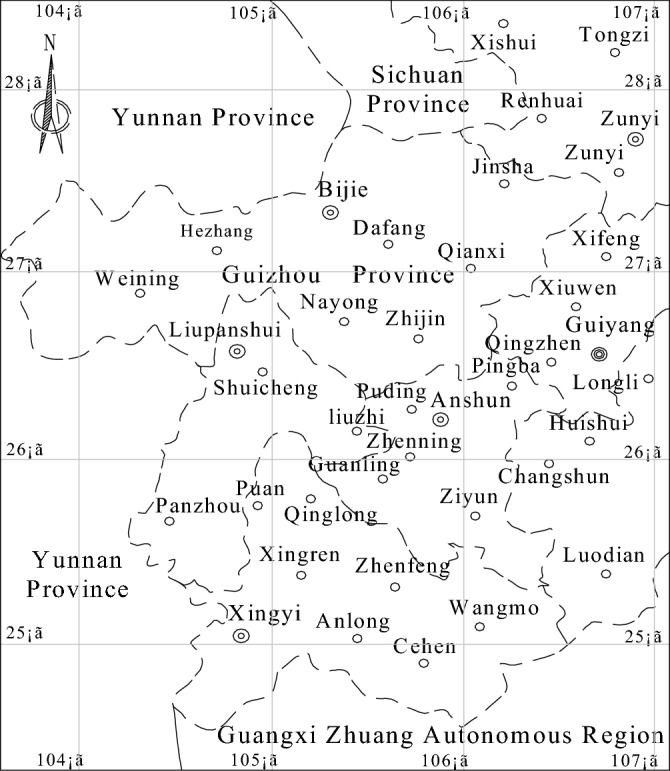
Distribution of the main coal-producing areas in Guizhou (according to the administrative division map of Guizhou Province (GS (2019) No. 2883)).

There are a large number of karst landforms that developed in different periods in the coal-bearing areas of Guizhou^15,16^ (see Fig. 1). The limestone strata exposed on the surface in the main coal-producing areas range from the Triassic Yelang Formation (Feixianguan Formation) (T_1_) to the Permian Maokou Formation (P_2_m). In karst landforms in different periods, weathering and dissolution can cause dissolution gaps and fissures that have developed to be buried deeply, providing good channels for the storage and infiltration of atmospheric precipitation^9,17,18^. The main coal-producing areas in Guizhou are distributed to the west of Zunyi city, Bijie city, and Liupanshui city, in Southwest Guizhou and to the west of Guiyang city^19^. As shown in Fig. 2, the coal seams of the Permian Longtan Formation between the Triassic Yelang Formation (Feixianguan Formation) (T_1_) and Permian Maokou Formation are the main mined coal seams, and each well field is located in an area with karst landforms.

### Geological and hydrogeological data collection

#### Weathering fissures and their depth of development

After the formation of coal seams in the mine field, due to weathering and tectonic movement, a large number of weathering fissures in rock strata have been formed on the surface. Under certain conditions, weathering fissures can not only store atmospheric precipitation but also become supply channels for atmospheric precipitation. The depth of weathering fissure development plays an important role in the supply of atmospheric precipitation to the deep stope. According to statistical data on weathering fissures (see Table 1), the burial depths of the weathering zone of exposed rocks on the surface are generally 10–30 m, up to 65 m^20–22^. The depth of local weathering fissure development in some mining areas, such as the Panzhou Datian coal mine in Liupangshui, Guizhou Province, is 65 m.

**Table 1:** Statistics of the depth of surface weathering fissure development.

Region	Name of coal mine	Depth of weathered fissure zone development (m)
Liupanshui	Datian coal mine	65
Bijie	GuanZhai coal mine	5–10
Yunnan, Guangxi and Guizhou border area		20–30
Linxian County, Shanxi		40–50

#### Mining fissures

**Figure 3:**
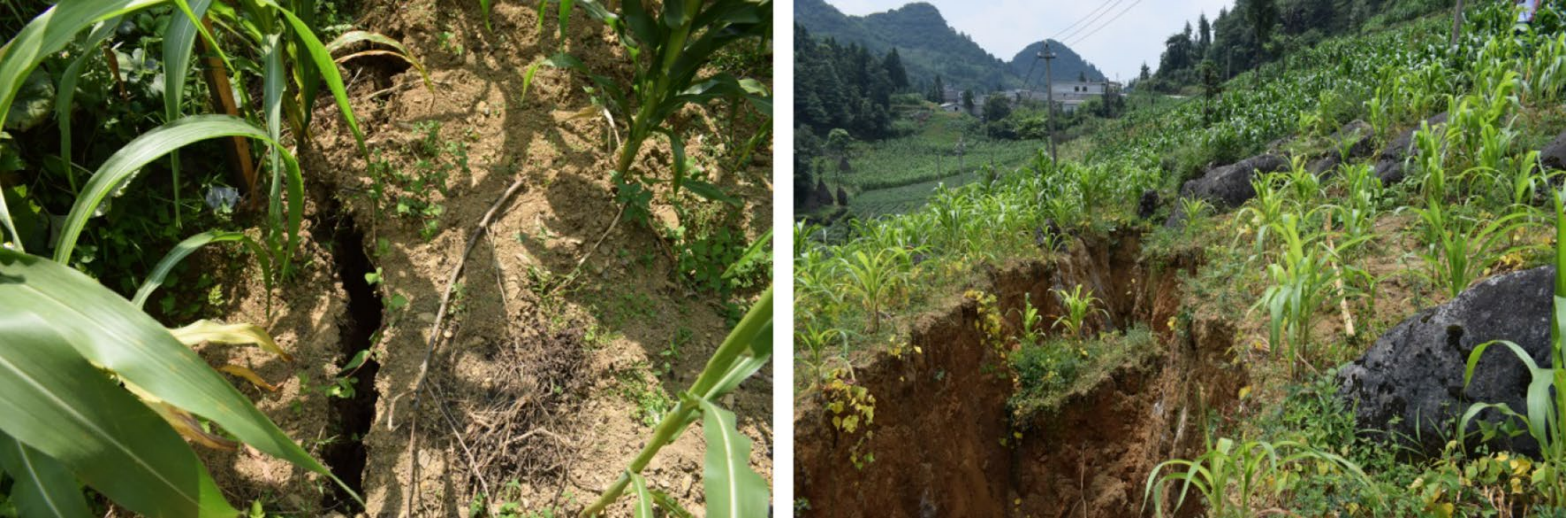
Surface fissures produced by coal mining.

According to the intensity of mining activities and the thickness of the coal seam, the height of the water-conducting fracture zone after coal seam mining differs^23^. When the heights of the leading and caving zones of the mining face reach the surface, weathered fracture zone or aquifer connected to the surface, atmospheric precipitation may replenish the mine through mining-induced fractures. Coal mining damages the surface and produces a large number of surface cracks^12,24,25^ (see Fig. 3), which is conducive to the collection and infiltration of atmospheric precipitation. Due to the large number of coal seam outcrops in Guizhou, most mines adopt inclined shaft development, and the first mining areas are mostly located near shallow outcrops. In the initial stage of mining, mines are affected by water-diverting fracture zones receiving atmospheric precipitation recharge.

#### Fault investigation

Fault structures are developed in the Guizhou karst area, and the exposed surface faults account for a large proportion of the faults exposed by exploration. According to the exploration data of the mine field in Zunyi city, Bijie city, Liupanshui city, Southwest Guizhou and Guiyang city, the proportion of large faults exposed by exploration in the mine field in this coal-producing area is 84.5%, and the proportion of various exposed faults is 69.7% (see Table 2). According to the statistical data, the faults exposed during mine field exploration and pumping tests show that 95% of the faults are not rich in water or do not conduct water. Due to later weathering and quaternary soil filling, most faults do not experience water diversion without being affected by mining damage. Some faults are activated due to later mining activities^26^, forming a water diversion channel that fills the working face or roadway with atmospheric precipitation through karst aquifers, the water-diverting fracture zone of the working face and surrounding rock fractures^5^. For example, in the four water inrush faults exposed at the working face of the No. 9 coal seam of the Guiyuan No. 2 well in Jinsha County, no water has been found during excavation. Some faults close to the surface can directly supply atmospheric precipitation to the mining face after the roadway is exposed or the working face is mined. In the process of mining the working face, the coal mine arranges special personnel to patrol the surface cracks. If surface cracks are found, timely measures are taken to block the cracks, reduce the area and quantity of the underground stope infiltrated by atmospheric precipitation, and reduce or eliminate the impact of atmospheric precipitation on safe production to increase the output of raw coal.

**Table 2:** Statistics of exposed surface faults.

Serial number	Region	Name of coal mine	Number of faults exposed in mine field exploration (pieces)		Number of exposed surfaces (pieces)		Percentage of exposed surface (%)	
			Number of large faults	Number of faults	Large fault	Fault	Large fault	Fault
1	Liupanshui	Tengqing coal mine	3	7	1	2	33.3	28.6
2		Faer coal mine	12	27	8	11	66.7	40.7
3		Dahebian coal mine	12	40	5	8	41.7	20.0
4		Dongguaao coal mine	2	8	2	7	100.0	87.5
5		Jinhe coal mine	4	23	3	10	75.0	43.5
6		Dongyu coal mine	10	15	8	9	80.0	60.0
7		Xiangxing coal mine	21	74	10	56	47.6	75.7
8		Shunyuan coal mine	11	49	11	49	100.0	100.0
9	Bijie	GuanZhai coal mine	10	24	7	15	70.0	62.5
10		Yangliu coal mine	31	56	29	35	93.5	62.5
11		Longhua coal mine	3	10	3	6	100.0	60.0
12		Tailai coal mine	3	6	3	4	100.0	66.7
13		Guiqing coal mine	4	11	3	4	75.0	36.4
14		Xintian coal mine	6	20	6	11	100.0	55.0
15		Buzuo mine field	17	83	13	41	76.5	49.4
16		Duijiangnan coal mine	17	20	15	14	88.2	70.0
17		Wufeng coal mine	2	14	2	9	100.0	64.3
19		Jiaotian coal mine	3	7	3	5	100.0	71.4
21		Liuhuangpo coal mine	8	10	8	9	100.0	90.0
22		Linhua coal mine	2	18	2	9	100.0	50.0
23		Dayun coal mine	4	8	4	6	100.0	75.0
24	Southwest Guizhou	Nuodong coal mine	3	17	1	2	33.3	11.8
25		Diguayi mine field	13	36	10	12	76.9	33.3
26		Happy mine field	15	21	13	16	86.7	76.2
27	Zunyi	Huaqiuer coal mine	20	31	11	22	55.0	71.0
28	Anshun	Pusheng coal mine	9	12	9	9	100.0	75.0
29		Angutielong coal mine	7	8	7	7	100.0	87.5
Total			252	655	197	388	78.2	59.2

#### Collapse column

Guizhou is dominated by karst landforms. Carbonate rocks in most of the well fields in the area are exposed in a large area, developing peak cluster depression landforms and internal troughs, depressions, sinkholes and funnels. For example, 62 sinkholes have developed on the surface of the Longhua coal mine in Qianxi County, 59 sinkholes have developed on the surface of the Linhua well field in Jinsha County and the nearby ground, and sinkholes and underground karst pipelines are relatively developed in the depression, and they are good supply channels for atmospheric precipitation^28^. The Longtan Formation, which is the main coal-bearing stratum in Guizhou, does not have the conditions for which giant thick limestone features, such as large karst caves, collapse columns and collapse pits, develop. A very thick limestone stratum is developed above the main coal-bearing stratum, but the bottom limit of karst development is the top of the Longtan Formation. Therefore, once the coal seam in the water-diverting fracture zone is connected with the overlying karst water diversion channel, atmospheric precipitation can replenish the mine. To the east of Bijie and Anshun, due to the development of the Maokou Formation limestone at the bottom of the main coal-bearing strata, the collapse column that developed in the Maokou Formation is connected with the karst channel above the coal measure strata (for example, two collapse columns were found underground in the Xing'an coal mine in Jinsha County, and four collapse columns were found in the Longhua coal mine). After comprehensive measures are taken to determine the hydrogeological conditions of the collapse column, direct mining can be adopted to reduce the impact of the collapse column on production and increase the coal output through the collapse column or by retaining the coal column. For example, the Xing'an coal mine in Jinsha County took the measure of directly passing through the collapse column after it was proven that the upper limit of the collapse column of the 21,307 working face was in the coal measure stratum; this was done to avoid skipping the mining of the working face, which would have affected production for 2 months.

#### Karst development

In the main coal-producing areas in Bijie, Zunyi, Guiyang and east of southwest Guizhou, a stable layer of Changxing Formation limestone is present above the Longtan Formation, which is the main coal measure stratum in the karst area. The thickness of this limestone layer are 32.6–48.8 m, the average thickness is 40.3 m, and the average distance to the nearest coal mine is 27.5 m. The aquifer is one of the main direct or indirect aquifers recharging the mine. During the exploration of each mine, the rate of water leakage in the Changxing Formation limestone reaches 30.4% (see Table 3). The karst fissures in the limestone are developed, and the connectivity is good. Atmospheric precipitation can be transported to the mining face through the area of karst development in the air-oxidizing zone of the Changxing Formation limestone outcrop to form a karst supply channel^9,28,29^. For the limestone channel in the Changxing Formation, the measures of sealing and filling the limestone outcrop on the surface and blocking the supply channel of atmospheric precipitation can reduce the number of mines that are affected by atmospheric precipitation, reduce the impact of atmospheric precipitation on the mining of the working face and improve the raw coal output of the mine.

**Table 3:** Statistics of the water leakage rate of limestone in the Changxing Formation.

Region	Name of coal mine	Number of exposed boreholes	Number of leaking holes	Water leakage rate (%)
Bijie	GuanZhai coal mine	44	6	13.6
	Gaoshan coal mine	12	2	16.7
	Tailai coal mine	8	3	37.5
	Qinglong coal mine	49	34	69.4
	Xintian coal mine	49	7	14.3
	Wufeng coal mine	75	10	13.3
	Dayun coal mine	47	24	51.1
	Linhua coal mine	41	8	19.5
Zunyi	Xinglong coal mine	34	13	38.2
Statistics		359	107	30.4

### Exploration of the relationship between mine water inflow and atmospheric precipitation

**Figure 4:**
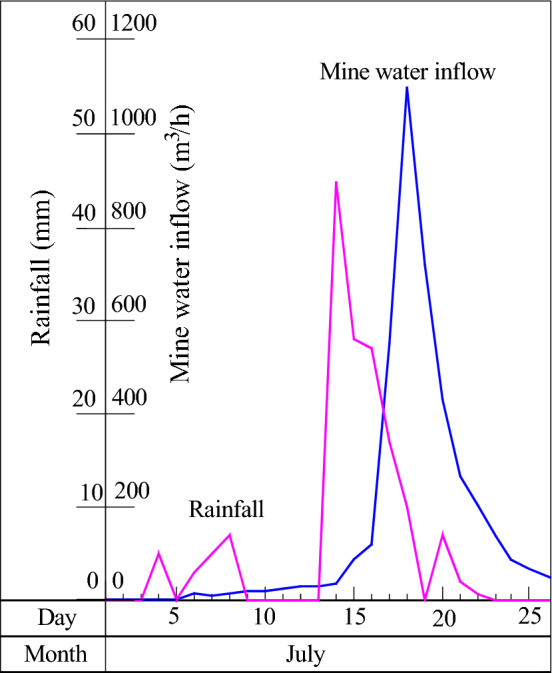
Distributions of water inflow and atmospheric precipitation in the No. 2608 working face of the Wufeng coal mine, Dafang County.

**Figure 5:**
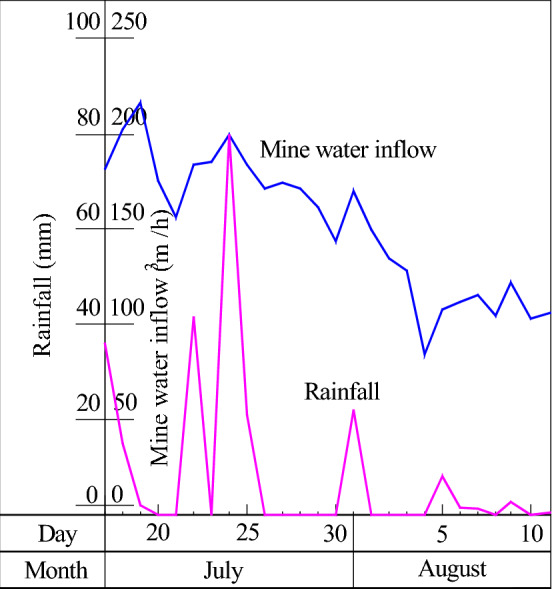
Distribution of water inflow and atmospheric precipitation after water outflow from the collapse column in the 1908 coal face of the Qianjin coal mine.

**Figure 6:**
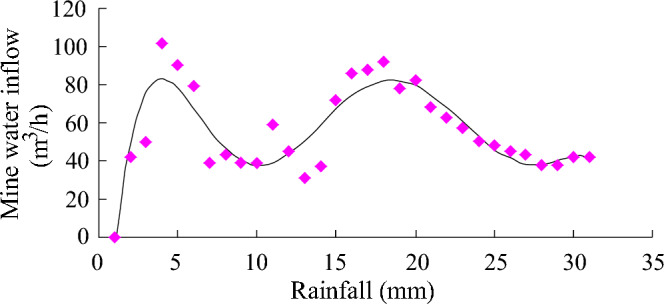
Regression curve of rainfall and water inflow in the 1402 coal face in the Xintian coal mine.

Based on the investigation of the mine that has an obvious correlation between mine water inflow and atmospheric precipitation, the water inflow and precipitation data of some typical mines were collected for different types of water-conducting channels, and the curves of the relationship between mine water inflow and atmospheric precipitation in fissure water-conducting channels, collapse column channels and karst channels were drawn, as shown in Table 4 and Fig. 4, Table 5 and Fig. 5, and Table 6 and Fig. 6, respectively. The curve of the relationship between mine water inflow and atmospheric precipitation under different water-conducting channel conditions indicates that atmospheric precipitation is the source of supply for mine water.

**Table 4:** Statistical table of water inflow and rainfall in the 2608 working face of the Wufeng coal mine in Dafang County, Bijie.

Date (month, day)	Rainfall (mm)	Water inflow (m^3^/h)	Date (month, day)	Rainfall (mm)	Water inflow (m^3^/h)
July 1	0	2	July 14	45	36
July 2	0	2	July 15	28	88
July 3	0	2	July 16	27	120
July 4	5	2	July 17	17	550
July 5	0	2	July 18	10	1100
July 6	3	15	July 19	0	720
July 7	5	10	July 20	7	430
July 8	7	15	July 21	2	266
July 9	0	20	July 22	0	203
July 10	3	20	July 23	0	138
July 11	0	25	July 27	0	49
July 12	0	30	July 31	0	49
July 13	0	30

**Table 5:** Statistical table of water inflow and rainfall in the 1908 working face of the Qianjin coal mine in Qianxi County, Bijie, after water inrush from the collapse column.

Date (month, day)	Rainfall (mm)	Water inflow (m^3^/h)	Date (month, day)	Rainfall (mm)	Water inflow (m^3^/h)
July 17	36	181.2	July 30	0	143.4
July 18	15	202	July 31	22	169.7
July 19	2	216	August 1	0	149.5
July 20	0	175	August 2	0	134.4
July 21	0	156	August 3	0	128
July 22	41.5	182.7	August 4	0	84
July 23	0	185	August 5	8.1	107.6
July 24	80	199.2	August 6	1.5	111.6
July 25	21	183.5	August 7	1.3	115.2
July 26	0	171.2	August 8	0	104.4
July 27	0	174.1	August 9	2.7	121.7
July 28	0	171	August 10	0	102.8
July 29	0	161.3	August 11	0.5	106

**Table 6:** Statistical table of water inflow and rainfall in the 1402 working face of the Xintian coal mine in Qianxi County, Bijie.

Date (month, day)	Rainfall (mm)	Water inflow (m^3^/h)	Date (month, day)	Rainfall (mm)	Water inflow (m^3^/h)
June 1	0	42	June 16	10	88
June 2	0	50	June 17	2	92
June 3	0	102	June 18	2	78
June 4	0	90	June 19	0	82
June 5	11	79	June 20	0	68
June 6	26	39	June 21	0	63
June 7	0	43	June 22	0	57
June 8	2	39	June 23	0	50
June 9	7	39	June 24	3	48
June 10	4	59	June 25	0	45
June 11	72	45	June 26	2	43
June 12	0	31	June 27	50.7	38
June 13	0	37	June 28	0	38
June 14	0	72	June 29	0	42
June 15	5	86	June 30	0	42

### Analysis of the chemical characteristics of mine water

After the increase in underground water inflow caused by rainfall in the mining area, samples were taken at the water outlet for the water quality test. For example, in the No. 2 well of the original coal mine in Jinsha County, the water quality at the outlet point in Changxing Formation limestone in the main shaft and the water inrush point of the working face is HCO_2_·SO_4_-Na-type water. The inrushing water contains nitride and fluoride, and the water source is supplied by surface atmospheric precipitation. The Linhua coal mine contains HCO_2_-Na-type water after the working effluent is removed. The water quality test results show that atmospheric precipitation is the source of water for the mine water.

## Results

Through investigating cracks, faults, collapse columns, and karst, as well as determining mine recharge water sources, this project has achieved the following research results.

### Channel type for the mine water supplied by atmospheric precipitation

#### Fissure passage

Weathering fissure infiltration channel.

Generally, the burial depths of the weathering zone of surface-exposed rock prior to the start of mining activities in coal mines are 10–30 m^17,18^, while the depth of local weathering fissure development in some mining areas, such as the Datian coal mine in Panzhou, is 65 m. Notably, weathering fissures contain water, phreatic water is present in the vadose zone, and even confined water is present in the low-lying areas of the mountains. On the other hand, the spring water level may have seasonal changes. After starting mining activity, atmospheric precipitation can supply water to the mine through weathering fractures. For example, the water inflow in the auxiliary shaft adit of the Wufeng coal mine in the Bijie area of Guizhou Province may exceed 13 m^3^/h in the rainy season, while it is only 3 m^3^/h in the dry season. Once the weathering zone is directly or indirectly connected to the water-conducting fracture zone, atmospheric precipitation can supply the mine.

Mining-induced fissure infiltration channel.

According to the intensity of mining activities and the thickness of the coal seam, the height of the water-conducting fracture zone after coal mining can differ. When the height of the guiding zone, the weathered fracture zone, or the aquifer reaches the surface, atmospheric precipitation may supply the mine through mining-induced fractures. Figure 3 shows that coal mining may result in a large number of surface fractures, which is conducive to the collection and infiltration of atmospheric precipitation. Due to a large number of coal seam outcrops in Guizhou, inclined shaft development has been adopted in most coal mines. The initial mining areas are typically near shallow outcrops. In the early stage of mining, the water-diverting fracture zone is affected by the supply of atmospheric precipitation. For example, the nearest distance between the 10,501 working face of the Jiaxing coal mine in Nayong County and the surface is 95 m. In this case study, the maximum water inflow during rainy seasons after mining reaches 51 m^3^/h, while the water inflow during dry seasons decreases to 3 m^3^/h. Then, it becomes stable at 2 m^3^/h. As another example, the No. 6 coal seam of the Shengan coal mine in Jinsha County is 162 m away from the nearest surface. In this case study, the maximum water inflow is 69 m^3^/h in rainy seasons, while it decreases to 5 m^3^/h in dry seasons.

#### Water-conducting faults

Fault structures are present in the Guizhou karst area, and the exposed surface faults account for a large proportion of the faults exposed by exploration (see Table 2). Some faults have been activated due to later mining activities^26,27^, forming water-diversion channels that fill the working face or roadway with atmospheric precipitation through karst aquifers, working face water-diverting fracture zones, surrounding rock fractures, etc. Some faults close to the surface can supply atmospheric precipitation directly to the mining face after the roadway is exposed or the working face is mined. For example, during the rainy season, the 10,403 fully mechanized mining face of the Shiqiao coal mine in Qianxi County is located at the footwall of a reverse fault with a drop greater than 30 m. After the working face is pushed 23 m, the working face produces water, with a maximum water yield of 522 m^3^/h. The water source is the atmospheric precipitation collected in a low-lying place 2 km away from the working face. Atmospheric precipitation penetrates into the underground working face through a reverse fault, resulting in an accident due to a flooded surface. After several consecutive days of rainfall, the water inflow in the working face rapidly decreases to less than 15 m^3^/h, and the water inflow in the goaf is 3 m^3^/h after the working face is mined.

#### Water diversion collapse column

Sinkholes and underground karst pipelines are relatively developed in karst landforms in Guizhou, and they are good supply channels for atmospheric precipitation^28^. The development of karst in the Maokou Formation limestone (P_2_m) in the extremely thick layer at the bottom boundary of the coal measure stratum provides good conditions for the development of the collapse column. When the collapse column develops above the top plate of the coal measure stratum, it is connected with the sinkhole at the surface, giving it the conditions for atmospheric precipitation to replenish the mine. After the collapse column is destroyed by coal mining, an initial small collapse column that lacks water conduction forms a channel for atmospheric precipitation to replenish the mine. For example, in the 1908 working face of the Qianjin coal mine in Qianxi County, a collapse column connected with the ground karst stratum was exposed during the rainy season (the long axis of the collapse column is 22.4 m, and the short axis is 15.5 m). When the working face passes through the collapse column, the collapse column has no water outflow. After the working face is pushed through the column, the roof falls, and the column is loose, resulting in atmospheric precipitation infiltrating into the shaft through this channel; this in turn results in water inrush in the working face, with the maximum water volume reaching 247 m^3^/h. After ground treatment, after the mining of the working face passes through the collapse column, the water inflow of the working face gradually decreases, and then the water inflow in the goaf is maintained at approximately 4 m^3^/h.

#### Karst channel

**Figure 7:**
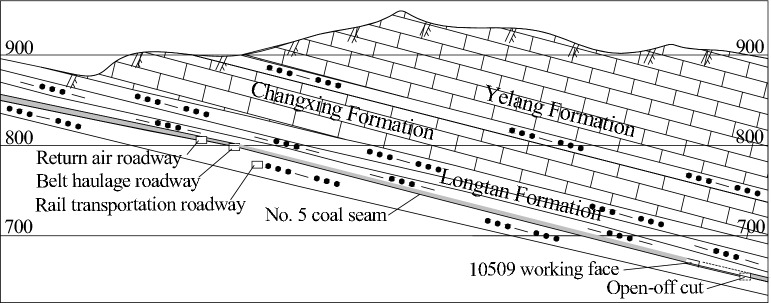
Profile of the precipitation recharge in the Jiuyuan coal mine, Jinsha County.

In the main coal-producing areas in Bijie, Zunyi, Guiyang and east of southwest Guizhou, a stable layer of Changxing Formation limestone is developed above the Longtan Formation, which is the main coal measure stratum in the karst area. The thicknesses of this limestone layer are 32.5–48.6 m, the average thickness is 39.5 m, and the average distance of the nearest coal mine is 27.4 m. The aquifer is one of the main direct or indirect aquifers recharging the mine. The aquifer in each mine field is mostly exposed on the surface in strips. Oblique fractures and vertical fractures are mostly developed in the rock stratum that was exposed by drilling. Most of the fracture surfaces are filled with vein calcite, and the widths of the fracture surfaces are different. There are occasional dissolution fractures or dissolution pores. Water rust is often seen in the dissolution fractures, most of the dissolution pores are filled with calcite crystals, and the rocks containing leakage holes are relatively broken. These features can become channels for atmospheric precipitation to replenish the mine stope. Atmospheric precipitation can be transported to the mining face through the area of karst development of the air-oxidizing zone of the Changxing Formation limestone outcrop, forming a karst replenishment channel^29^. The No. 5 coal seam in the Jiuyuan coal mine in Jinsha County is 106–251 m away from the surface and 37.4 m away from the Changxing Formation limestone. The working face is mined up the mountain, as shown in Fig. 7. The outcropping limestone of the Changxing Formation is supplied by atmospheric precipitation. The maximum water inflow in the goaf is 85 m^3^/h in the rainy season and 7 m^3^/h in the dry season.

### Characteristics of atmospheric precipitation recharge

#### Surface infiltration

**Figure 8:**
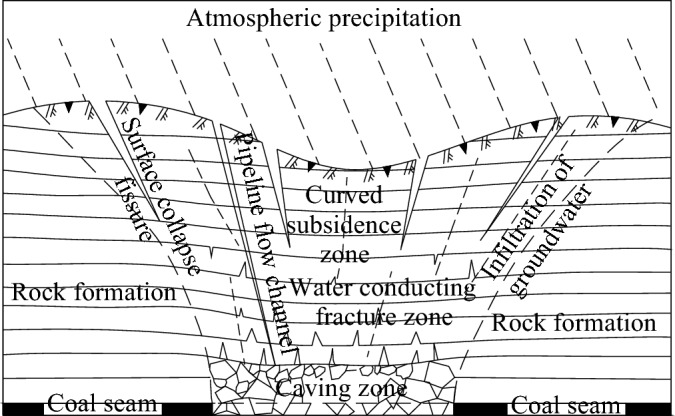
Pattern of large-area infiltration of atmospheric precipitation into mine stopes.

After the mining of coal seams in the mine is completed, a large area of surface subsidence causes the development of a surface fracture zone, with fracture widths ranging from 0 to 3 m (see Fig. 3) and depths ranging from tens of metres to tens of metres, forming good atmospheric precipitation infiltration channels. A large area of surface fracture development forms good conditions for the infiltration of atmospheric precipitation underground. Through atmospheric precipitation after large-scale infiltration, it gradually collects and seeps into the working face and flows into the mine in the working face or goaf (as shown in Fig. 8), resulting in dripping water on the roof of the working face, and drenching water and water gushing from the goaf, and the amount of water gushing into the mine increases. Under surface infiltration conditions, because the topsoil and aquifer need a certain water-filling saturation period, the underground water inflow increases significantly after several days of continuous precipitation. In the Guizhou karst mountainous area, in most mines, the infiltration of atmospheric precipitation at the surface causes an increase in water inflow to the mine and even causes accidents resulting from flooded working faces and flooded mining areas^1^. For example, the distance between working face 2608 in the Wufeng coal mine in Dafang County and the surface is less than 100 m, and the ground fissures in the goaf after mining receive a large range of atmospheric precipitation supply. The water inflow of the working face increases rapidly (see Fig. 4), resulting in flooding of the working face and the lower third of the mining area.

The lag of the supply of atmospheric precipitation to the mine is affected by the distance between the stope and the surface. Generally, the shallower the coal seam is, the more it is affected by atmospheric precipitation, and the farther it is from the surface, the more the supply of atmospheric precipitation to the mine lags. In mines with obvious atmospheric precipitation recharge, the lag time of surface infiltration recharge mines is generally 1–4 days^30^, such as in the Sheng'an coal mine in Jinsha County and the Wufeng coal mine mentioned above. For some coal mines with deep stopes, the lag period for mine water supplied by atmospheric precipitation is long. For example, in the Nuodong coal mine in southwest Guizhou, the coal seams in the mine are nearly horizontal, with an average distance of 304 m from the surface. The water inflow of the mine begins to increase (decrease) and lags behind the rainy season (dry season) by 3–4 months.

The areal infiltration is obviously affected by the burial depth of the coal seam and the characteristics of the combined lithology. The amount of water supplied by atmospheric precipitation to the mining face at the same depth is not consistent. In some mines, when hard rock layers such as limestone are used as the key layers in the coal seam roof, the combination of soft and hard rocks leads to the following in some mines: when the mining distance from the surface is less than 150 m, atmospheric precipitation has no impact on goaf filling. For example, the working face of the No. 15 coal seam in the Xing'an coal mine in Jinsha County is approximately 100 m away from the surface, and the upper strata are a combination of limestone, sandstone, sandy mudstone, mudstone and coal seam. The water inflow in the goaf of the working face is generally less than 2 m^3^/h, and the impact of atmospheric precipitation on the water inflow of the mine is not obvious. In the Gaoshan coal mine in western Guizhou and the Jiaozishan coal mine in Anshun, the mine water inflow during the mining period of the shallow working face is obviously affected by atmospheric precipitation, and the impact at depth is gradually reduced. The coal seam mined in the Daheban coal mine in Liupanshui is 314–677 m from the ground. At shallow depths, the amount of atmospheric precipitation in the rainy season is 2.2 times that in the dry season and 1.3 times that at depth.

#### Pipeline flow characteristics

Atmospheric precipitation supply channels that have the characteristics of pipeline flow channels are generally composed of collapse columns, karst channels and fault fracture zones^8,9,29^. Due to the smooth water channel, the underground recharge time of atmospheric precipitation is relatively short, and atmospheric precipitation has the characteristics of concentrated water inflow, large water inflow and obvious fluctuation with rainfall, as shown in Fig. 5. This kind of water diversion channel easily causes the mine working face or mining area to be flooded, which seriously affects safe mine production. In this kind of water diversion channel, atmospheric precipitation can generally be replenished to the mine stope within 2–24 h. For example, the water inrush of the 10,403 working face of the Shiqiao coal mine in Qianxi County reached 300 m^3^/h, and the water inrush reached the underground working face 2 h after rainfall reached the ground.

#### Characteristics of layered recharge

The conditions for the supply of atmospheric precipitation to the mine through a specific aquifer are relatively harsh. The aquifer need not only have a surface outcrop or be close to the surface to easily accept the supply of atmospheric precipitation but also have a water diversion channel. Moreover, the aquifer can supply the mine directly or indirectly, and the limestone in this layer has become a layered supply channel for the mine. The thickness of coal seam 4 in the Xintian coal mine of Qianxi County is 2.8 m, and it is 27 m from the overlying Changxing Formation limestone (see Table 2). The Changxing Formation limestone is within the water-diverting fracture zone of coal seam 4 and is the indirect water-filling aquifer of the mine. During the mining of working face 1402 of the mine, the water inflow follows the working face and has an obvious correlation with atmospheric precipitation (as shown in Fig. 6). According to Table 6, the regression equation between the water inflow and atmospheric precipitation is

1 $${\text{y }} = \, - 0.0000{\text{3x}}^{{6}} + \, 0.00{\text{38x}}^{{5}} - \, 0.{\text{1578x}}^{{4}} + { 3}.{\text{167x}}^{{3}} - { 3}0.{\text{889x}}^{{2}} + { 13}0.{\text{74x }} - { 111}.{8}.$$

In the formula: y—water inflow (m^3^/h); x—Rainfall (mm).

The correlation coefficient r reaches 0.88, and there is an obvious correlation between mine water inflow and atmospheric precipitation.

The limestone in the range of mining damage, especially the limestone aquifer of the Changxing Formation, is not only the main aquifer of the Longtan Formation coal measure strata but also the channel for atmospheric precipitation supply. For mines receiving limestone channel recharge, the correlation between atmospheric precipitation recharge is obvious, and the rainwater recharge underground generally occurs within 2 days.

### Self-healing characteristics of the supply channel

In the Guizhou Karst mining area, the surface is mostly covered by loess layers with different thicknesses in the Quaternary system. Due to the scouring by continuous rainfall and the disturbance to the surface stratum by mining, a large amount of rainwater carries loess particles into the rock fissures and flows into the mine stope, resulting in a yellow colour of inflowing mine water in the goaf and roadway of many mines. Loess particles flowing into the pores of rock strata during the rainy season gradually cause pore blockage as the precipitation decreases during the dry season. This phenomenon is confirmed by the fact that the cracks in the weathered rock exposed by the shaft during the construction of many mines in Guizhou are basically filled with loess. As the fissures are filled with precipitated soil, the mine water inflow caused by various channels gradually decreases, and some goafs do not flow out after 3–5 years. In the first rainy season after mining the 1501 working face of the Jiaxing coal mine in Nayong County, the water inflow in the goaf of the working face reached 53 m^3^/h, which was significantly reduced to 10 m^3^/h in the second rainy season and then stabilized at 2 m^3^/h in the rainy season. The water inflow in the goaf (145 m^3^/h in the initial rainy season) and the final goaf of the 1614 working face of the Wufeng coal mine in Dafang County decreased to less than 10 m^3^/h in the rainy season.

### Seasonal recharge characteristics of atmospheric precipitation

**Figure 9:**
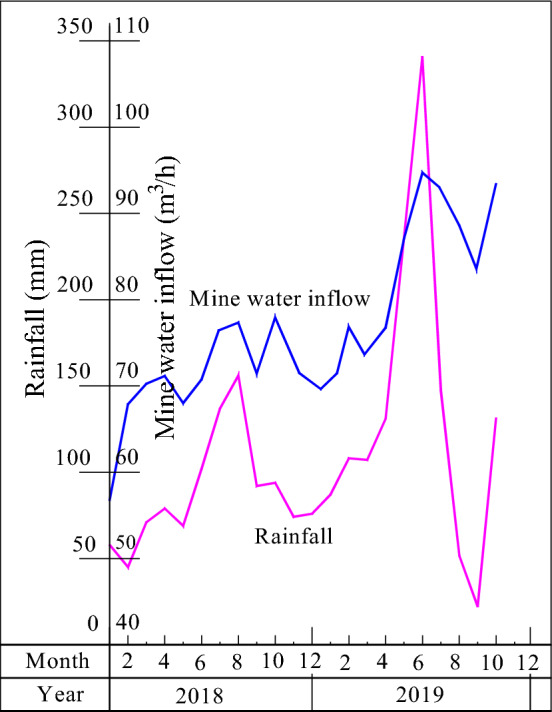
Distribution of the mine water inflow and atmospheric precipitation in the Anshun coal mine.

The seasonal variation in mine water inflow in Guizhou is obvious, as shown in Table 7 and Fig. 9. The main coal-producing areas in Guizhou are located in areas of karst development, which have rainy and dry seasons. During the rainy season, the atmospheric precipitation obviously replenishes the mine, and the mine water inflow obviously increases. In the dry season, the atmospheric precipitation is relatively low, and the mine water inflow is significantly reduced. According to statistical data from different mines, the water inflow of mines in the rainy season is generally 1.2–12 times that in the dry season, with an average of 1.9 times. This feature is obviously different from that of the North China-type stratigraphic mining area covered by extremely thick Quaternary loose layered sediments. According to this feature, the mine can reasonably adjust the mining time, organize the production in the dry season, and reduce the impact of atmospheric precipitation on mine safety to improve coal output.

**Table 7:** Statistical table of water inflow and rainfall in the Anshun coal mine, Xixiu District, Anshun.

Date (month, year)	Rainfall (mm)	Water inflow (m^3^/h)	Date (month, year)	Rainfall (mm)	Water inflow (m^3^/h)
January 2018	58.0	56.7	December 2018	75.9	77.8
February 2018	45.0	67.9	January 2019	87.0	78.2
March 2018	71.1	70.3	February 2019	108.1	76.7
April 2018	78.9	71.1	March 2019	107.2	77.3
May 2018	68.9	68.0	April 2019	131.2	76.8
June 2018	102.1	70.7	May 2019	235.6	87.1
July 2018	136.9	77.3	June 2019	340.2	94.4
August 2018	155.8	77.2	July 2019	147.1	99.1
September 2018	92.3	71.4	August 2019	51.8	91.1
October 2018	93.8	77.9	September 2019	22.2	89.5
November 2018	74.2	77.8	October 2019	179.3	81.3

## Discussion

### Determination of channels supplying atmospheric precipitation

**Figure 10:**
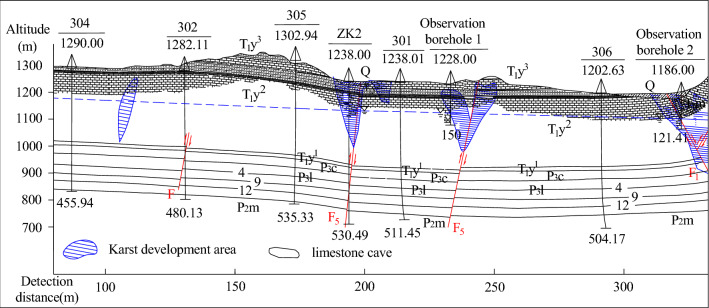
Geological profile inverted by the magnetotelluric method.

When it was discovered that atmospheric precipitation supplied the mine through faults, collapse columns, aquifers and other channels, some mines carried out special research on treating for water damage. Personnel at the Xintian coal mine have detected the depth of surface karst development in the mine field by using the controllable audio frequency magnetotelluric method. The measured depth of karst development is 298 m, and the depth of karst development revealed by drilling is 412.82 m (see Fig. 10). The drainage test determined the water barrier boundary of the mine field and the water conductivity (water barrier) of the fault and verified that atmospheric precipitation replenished the mine stope through the Changxing Formation limestone through a chemical reagent tracking test.

After water inrush accidents caused by atmospheric precipitation, the personnel at most mines carried out supplementary exploration of ground hydrogeology, further explored the hydrogeological conditions of the mine by using drilling and geophysical exploration technology, and took water samples at the water outlet for testing; they verified that atmospheric precipitation supplied the mine through the Changxing Formation limestone and analysed the water inrush pattern. By reasonably controlling the mining speed, greater impacts of atmospheric precipitation and water damage on mine safety during production are avoided. Personnel at the Qianjin coal mine used a hydrogeological map combined with electrical exploration to determine the area of precipitation and collapse in the surface catchment area. During the mining of the No. 4 coal seam overlying the 1908 water outlet working face, there was no atmospheric precipitation supplying the working face, ensuring the safe extraction of 190,000 tons of raw coal in the mine.

### Surface sealing fissures

The method of atmospheric precipitation supplied to coal mines in Guizhou is mainly lateral infiltration. The amount of infiltrated atmospheric precipitation is affected by topography, overburden coverage, overburden thickness, rainfall continuity and rainfall intensity and the influence of comprehensive factors, such as the degree of permeability (flow) fracture development, water content in fractures and water content in the vadose zone^31,32^. According to the relationship between atmospheric precipitation and mine water inflow, combined with the scale of ground fissure development, overburden thickness and topographic characteristics resulting from mining activities, different mines have taken different measures to prevent and control the fissure channels that supply atmospheric precipitation.

**Figure 11:**
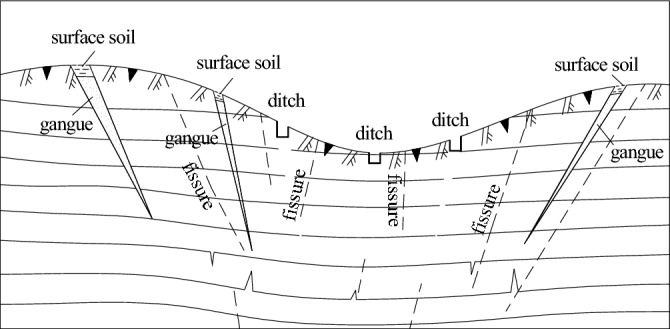
Schematic diagram of the treatment project of the fissure channel of infiltration by atmospheric precipitation.

For cracks with large ground openings, most mines use gangue filling to seal them. First, they are filled and compacted with crushed gangue and then sealed with topsoil 300 mm above the surface of the gangue (see Fig. 11). For small fractures, the surface soil near the fractures is used for filling, such as in the Anshun coal mine and Jiaozi Snow Mountain coal mine in Anshun city and the Xing'an coal mine in Jinsha County.

To prevent rainwater that accumulates on the surface from seeping into the ground again during the runoff process, some mines have constructed intercepting ditches near the hillside and ditch bottom to intercept precipitation to low-lying areas and then use drainage ditches to concentrate and discharge the water to noninfiltration areas, thereby flowing out of the mining area.

Disturbing the surface soil, utilizing rainwater erosion to bring small soil particles into small cracks, filling small cracks, blocking small water channels, and disrupting the infiltration pathways of small cracks are methods for preventing rainwater infiltration. After the implementation of these methods, the water inflow in goaf areas affected by atmospheric precipitation in the Wufeng coal mine in Dafang County and Jiaxing coal mine in Nayong County have significantly decreased yearly.

### Reasonable arrangement of the mining time of the working face

Atmospheric precipitation has seasonal characteristics. In some mines in the Guizhou karst mountainous area, the working face of shallow mining is less than 100 m from the surface. During the rainy season, atmospheric precipitation from mining is easily replenished to the underground working face through various cracks and limestone channels.

Due to the significant decrease in atmospheric precipitation during the dry season, there may be no precipitation for a long time. Although mining activities during the mining period of working faces close to the surface also lead to the development of ground fissures, underground mining sites are basically not affected by atmospheric precipitation recharge. Based on the seasonality of precipitation, some mines have reasonably adjusted their mining layout and production plan and have mined more shallowly buried working faces during dry seasons to avoid the impact of atmospheric precipitation on safe production, such as in the Sheng'an coal mine in Jinsha County. By mining the 10,903 working face during the dry season, the average monthly coal production of the Sheng’an coal mine is 33,000 tons, which is much higher than the average monthly production of 18,000 tons during the mining of the 10,901 working face in the rainy season.

For working faces that are mined during the dry season, the mine can use the time before the rainy season to treat the surface fissures of the mining area and combine this method with the disturbance and filling effects that spring farming has on the surface loess to effectively fill the mining fissures. In the rainy season in the second year of mining, the water inflow in the goaf is generally not large, which can significantly reduce the impact of rainfall on mine safety production.

### Improving the mine drainage capacity

In the Guizhou karst area, the water flowing into the mine infiltrated by atmospheric precipitation is not calculated when estimating the mine water inflow during the exploration of each mine field. The estimated mine water inflow in the exploration report is the water flowing into the mine replenished by the aquifer. During the construction of the mine, the capacity of the drainage system is selected and equipped according to the water inflow provided in the mine exploration report. During the production of the mine, because the coal measure strata in the Guizhou karst area are exposed to the surface, the shallow mining face is close to the surface, and atmospheric precipitation is supplied to the underground stope through the above channels, such as weathering fissures and mining fissures of the working face. The water flowing into the mine is far greater than the expected water inflow before the construction of the mine. As a result, the drainage equipment selected in advance cannot meet the needs of safe mining production, and most mines have upgraded the drainage system during production, such as the Wufeng Second Mine in Dafang County, Shiqiao coal mine in Qianxi city, and Min'an coal mine in Jinsha County.

After the drainage system is renovated, the drainage capacity of the mine generally increases by 50–200%. For example, the drainage capacity of a single drainage pump in the Sheng'an coal mine in Jinsha County is 155 m^3^/h, and the drainage capacity of a single pump after the renovation reaches 280 m^3^/h. Two φ108 mm drainage pipelines have been transformed into φ200 drainage pipelines. The Linhua coal mine and Tenglong coal mine in Jinsha County have also installed a submersible pump drainage system that is directly controlled by a ground power supply and has a drainage capacity that is no less than the maximum mine water inflow.

## Conclusions

To the best of our knowledge, this article studied the channels and types of atmospheric precipitation supplied to mining areas in areas of karst landform development for the first time and systematically elaborated on the characteristics of atmospheric precipitation supplied by each supply channel type; this study might fill the gap in the related field in the current literature. The main research findings of this article are as follows:

1. In mining areas with surface karst development, the main channels that supply atmospheric precipitation to mines are surface weathering fractures, mining fractures, faults, water-conducting collapse columns, limestone karst channels, etc.
2. Different water-conducting channels have different characteristics of atmospheric precipitation recharge. With a wide range of recharge of atmospheric precipitation by surface infiltration, the time it takes for infiltration to recharge the mines lags behind atmospheric precipitation by 1–4 days. Atmospheric precipitation can be supplied to the underground mining stope within 2–4 h through pipeline flow supply channels, and the amount supplied is large. Atmospheric precipitation can be replenished to the mining stope within 2 days through layered recharge channels. After being filled with surface soil particles, various types of channels supplying atmospheric precipitation have the characteristics of automatic sealing and self-healing.
3. Based on the conditions of fissure channels and the large-scale infiltration of atmospheric precipitation, the map of surface infiltration via atmospheric precipitation has been improved. A regression equation between mine water inflow and rainfall during the infiltration of atmospheric precipitation in layers has been established, providing a new method for predicting mine water inflow.

The results of this study may provide a theoretical basis and practical experience for further research on the prevention and control of atmospheric precipitation and water damage in areas with karst landform development. The research results provide a theoretical basis and practical experience for changing the singular method of passive drainage to control water disasters in mines and will help to promote research on the mechanism of atmospheric precipitation infiltration into mines in karst landform areas.

## Data Availability

The authors of the article declares that all data generated or analyzed during this study period is within the article.
